# Recent advances, comparative performance, and data requirements of artificial intelligence-assisted technologies for the diagnosis of gastric cancer

**DOI:** 10.3389/fgstr.2026.1878353

**Published:** 2026-07-22

**Authors:** Ziyang Zhang, Zhaorui Liu, Xin Zhang, Keke Lv, Yayu Huang, Chenxi Zheng, Tianlin He

**Affiliations:** Changhai Hospital, The First Affiliated Hospital of Naval Medical University, Shanghai, China

**Keywords:** artificial intelligence, clinical decision support system, digital pathology, early diagnosis, gastric cancer, medical imaging, model comparison, multimodal fusion

## Abstract

Early diagnosis of gastric cancer is critical for improving prognosis and increasing the 5-year survival rate. However, gastroscopy and pathological examination are invasive, resource-dependent, and associated with low adherence, while conventional serological and imaging screening methods have limited sensitivity for early lesions. In recent years, artificial intelligence (AI), especially deep learning, has improved the efficiency and accuracy of real-time endoscopic detection, digital pathological recognition, non-contrast CT-based opportunistic screening, and multimodal diagnostic decision support. This review summarizes recent advances in AI-assisted technologies for gastric cancer diagnosis and, in response to the need for a clearer methodological and quantitative framework, compares representative models according to data modality, model architecture, dataset size, validation strategy, and reported diagnostic performance, including AUC, accuracy, sensitivity, specificity, precision/positive predictive value (PPV), and F1 score when available. We further discuss how data size, modality diversity, annotation granularity, class imbalance, and missing modalities influence model performance in real-world multicenter settings. Finally, we propose a structured multimodal AI-clinical decision support system (AI-CDSS) framework integrating CT imaging, endoscopic images and videos, whole-slide pathological images, serological indicators, molecular biomarkers, and clinical reports. Although AI-assisted diagnosis has shown considerable promise, clinical translation still requires prospective multicenter validation, standardized reporting of performance metrics, interpretable model outputs, privacy-preserving data governance, and regulatory-compliant deployment.

## 1 Introduction

Gastric cancer (GC) originates from gastric epithelial cells. As of 2022, gastric cancer ranked fifth worldwide in both incidence and mortality among malignant tumors, with approximately 970,000 new cases and 660,000 deaths, posing a serious threat to human health (1). In China, approximately 360,000 new cases of gastric cancer and 260,000 related deaths occur each year, making it the third leading cause of cancer-related death (2). More than 60% of Chinese patients with gastric cancer are already at an advanced stage at the time of diagnosis, a situation closely associated with a relatively low 5-year survival rate of approximately 35% to 40% (3). In sharp contrast, the 5-year survival rate of patients with early gastric cancer can exceed 90% after curative treatment, such as surgery or endoscopic therapy (4). Therefore, early detection, early diagnosis, and early treatment constitute the core strategy for reducing gastric cancer mortality (5).

The current core diagnostic framework for gastric cancer comprises four major components: screening, imaging examination, endoscopic examination, and pathological diagnosis. Among these, gastroscopy combined with pathological biopsy remains the “gold standard”, as it can determine the histological type, degree of differentiation, and depth of invasion of the tumor. However, this examination requires transoral insertion of an endoscope and biopsy sampling; it is therefore an invasive procedure that must be performed by specialized physicians. It can easily cause discomfort such as nausea and vomiting, and population adherence is less than 30% (6, 7), making large-scale screening difficult to implement. Tumor marker testing, such as CEA and CA724, and serum pepsinogen (PG) testing are noninvasive, but their sensitivity and specificity are limited. They are suitable only for risk stratification and cannot provide a definitive diagnosis. Even for emerging liquid biopsy markers in recent years, such as exosomal lncRNAs, the results may still be affected by individual differences, disease stage, testing platform, and other factors; clinically, they are usually used only as auxiliary evidence (8) and cannot replace pathological diagnosis. Among imaging examinations, contrast-enhanced CT is mainly used for tumor staging. Non-contrast CT has traditionally been considered unsuitable for screening because gastric distension varies greatly, interference from gastric contents is common, and early lesions are subtle (9, 10). In addition, uneven endoscopic diagnostic proficiency in primary medical institutions and imbalanced allocation of medical resources further aggravate the challenges of early diagnosis. Therefore, efficient, convenient, and broadly applicable technological breakthroughs are urgently needed.

Artificial intelligence, particularly the deep learning (DL) branch, can automatically learn complex and subtle feature patterns from massive datasets through multilayer neural network models. It has demonstrated performance surpassing that of human experts in fields such as image recognition (11) and has already played an important role in specific diseases, including pulmonary nodule detection and the diagnosis of diabetic retinopathy (12). Its core advantages include: ① quantitative analysis of subtle features that are difficult for the human eye to detect, thereby improving the detection rate of early lesions (11, 13); ② reduction of diagnostic subjectivity and realization of standardized assessment (14); ③ efficient processing of massive medical data, thereby alleviating physicians’ workload (15); ④ adaptability to multimodal data integration, enabling the construction of a comprehensive disease representation system (16).

The development of AI technologies in gastric cancer diagnosis has been driven by three major clinical needs. First, there is a need for large-scale early screening. Given China’s large population, the conventional gastroscopy-based model cannot achieve full coverage, creating an urgent demand for noninvasive and low-cost preliminary screening technologies. Second, there is a need for precise diagnosis. Early gastric cancer lesions often manifest as subtle mucosal changes and are easily missed by conventional methods; therefore, AI assistance is needed to improve the accuracy of feature recognition (17). Third, there is a need to balance medical resources. By empowering primary medical institutions with AI, regional disparities in diagnostic capabilities can be reduced. In recent years, with the acceleration of medical data digitization, improvements in computational power, and optimization of algorithms, AI-assisted diagnostic technologies for gastric cancer have entered a phase of rapid development. Several breakthrough studies have been published in leading journals such as *Nature Medicine*, laying the foundation for a transformation in the diagnostic paradigm.

Therefore, this review aims to: ①summarize recent advances in AI-assisted imaging, endoscopic, pathological, and multimodal diagnosis of gastric cancer; ②compare representative AI models in terms of architecture, dataset size, validation design, and diagnostic performance; ③discuss how data size, modality diversity, annotation quality, class imbalance, and missing modalities affect model performance; ④propose a structured multimodal AI-CDSS framework for future gastric cancer diagnostic studies.

## 2 Methods of literature search and data extraction

This narrative review was conducted to summarize recent advances in AI-assisted technologies for gastric cancer diagnosis, with emphasis on medical imaging, endoscopy, digital pathology, molecular biomarker prediction, and multimodal fusion. Literature was searched in PubMed, Web of Science, Embase, IEEE Xplore, and Google Scholar from January 2019 to May 2026. Search terms included “gastric cancer”, “artificial intelligence”, “deep learning”, “machine learning”, “endoscopy”, “computed tomography”, “digital pathology”, “whole-slide image”, “multimodal fusion”, “diagnosis”, “screening”, “staging”, “biomarker”, and “prognosis”.

Studies were prioritized if they reported AI or machine-learning models for gastric cancer detection, classification, invasion-depth assessment, lymph-node metastasis prediction, molecular biomarker prediction, prognosis prediction, or diagnostic decision support. Extracted information included data modality, dataset size, model architecture, annotation strategy, validation strategy, diagnostic task, AUC, accuracy, sensitivity, specificity, precision or PPV, negative predictive value (NPV), F1 score, and major clinical limitations. Because the purpose of the present article is a narrative and methodological review rather than a formal meta-analysis, pooled effect estimates were not calculated. When a metric was not reported in the original study, it is indicated as “NR” in the comparison table.

## 3 Recent advances in artificial intelligence-assisted imaging diagnosis of gastric cancer

Medical imaging is an important basis for gastric cancer screening and staging, encompassing multiple modalities such as CT, gastroscopy, and ultrasonography. The core application of AI in imaging diagnosis lies in the use of deep learning models to enable automatic lesion detection, segmentation, and benign-malignant differentiation. Among these applications, AI-based screening using non-contrast CT and AI-assisted diagnosis using endoscopic images have become research hotspots in recent years and have achieved paradigm-shifting advances.

### 3.1 Technological breakthroughs in AI-based early screening using non-contrast CT

Conventionally, non-contrast CT has not been incorporated into routine screening because its low tissue contrast and the complex anatomical structure of the stomach make it difficult to identify early gastric cancer lesions confined to the mucosal layer, where the mucosal thickness is approximately 0.7-1.5 mm (9). In 2025, a team from Zhejiang Cancer Hospital and other institutions published a study in *Nature Medicine* in which they developed GRAPE, an AI-based gastric cancer risk assessment pipeline using routine non-contrast CT. Its ability to identify gastric cancer, including some early T1/T2 cases, was validated in multicenter and real-world cohorts, and it outperformed radiologists in comparative image-reading analyses. This study provides evidence supporting the use of non-contrast CT for opportunistic screening of gastric cancer (18).

#### 3.1.1 Technical innovations of the GRAPE model

The GRAPE model adopts a three-dimensional deep learning framework, with the following key innovations: ① This study employed a multicenter research framework. During model development, a modeling cohort from two centers, comprising 3,470 gastric cancer cases and 3,250 non-gastric cancer cases, was used for training, providing sufficient annotated samples for model construction. Its generalizability was further validated using external multicenter cohorts. ② A “contrast-enhanced CT-non-contrast CT” cross-modal alignment strategy was adopted. Tumor regions on contrast-enhanced CT were manually delineated to guide the model in learning the corresponding subtle features on non-contrast CT, thereby addressing the challenge of tumor annotation on non-contrast CT. ③ A dual-branch segmentation-classification architecture with multilevel feature fusion was used to learn subtle three-dimensional morphological changes of the gastric wall, thereby improving the ability of non-contrast CT to identify early gastric cancer (18).

#### 3.1.2 Clinical validation and application value

The GRAPE model has demonstrated favorable clinical application potential in opportunistic screening for gastric cancer. In internal validation and external multicenter validation, the model achieved AUC values of 0.970 and 0.927, sensitivities of 85.1% and 81.7%, and specificities of 96.8% and 90.5%, respectively. In a real-world cohort comprising 78,593 consecutive non-contrast CT examinations from Zhejiang Cancer Hospital and two regional hospitals, the gastric cancer detection rates among high-risk individuals identified by GRAPE in the two regional hospitals were 24.49% and 17.65%, respectively, with estimated sensitivities of 88.7% and 91.9% and specificities of 95.1% and 86.1%, respectively. Among these cases, T1/T2-stage tumors accounted for 23.2% and 26.8%, respectively, suggesting that the model has substantial value for early diagnosis and early screening. More importantly, the model can identify cases initially missed by radiologists. In a retrospective analysis, it successfully detected cancer-related signals on non-contrast CT scans obtained 2–10 months before confirmed diagnosis in seven patients with gastric cancer. In one 45-year-old patient with locally advanced disease, a non-contrast CT scan performed six months earlier for pulmonary nodule assessment was flagged as high risk by the AI model, indicating that timely intervention might have provided an opportunity for earlier treatment (18).

From a methodological perspective, GRAPE illustrates that a 3D segmentation-classification pipeline with cross-modal supervision can be more suitable than conventional two-dimensional image classifiers for non-contrast CT screening, because it preserves volumetric gastric-wall morphology and allows weak lesion localization. However, before routine population-level deployment, future studies should report precision/PPV, false-positive workload, calibration, decision-curve analysis, referral-to-endoscopy yield, and cost-effectiveness under different screening thresholds.

Compared with conventional approaches, “non-contrast CT + AI” offers three major advantages. ① In terms of convenience and noninvasiveness, non-contrast CT is often already routinely acquired for clinical examinations or health check-ups, allowing opportunistic screening without additional invasive procedures. By contrast, multiple population-based studies have shown that participation rates in gastroscopic screening are often below 30% (6, 7). ② In terms of cost control, the examination cost is only approximately one-third that of contrast-enhanced CT. It can be embedded into existing workflows without the need for additional equipment, thereby offering health economic advantages. ③ In terms of broad applicability, it provides a feasible screening strategy for populations who cannot tolerate gastroscopy, such as elderly individuals and patients with cardiopulmonary diseases.

### 3.2 AI-assisted diagnostic technologies based on endoscopic images

Gastroscopy is a core modality for the diagnosis of gastric cancer. However, early lesions often present as flat elevations, slight depressions, or changes in mucosal color, making them difficult to distinguish from benign lesions such as gastritis. Diagnostic accuracy therefore depends heavily on physicians’ experience (19). Studies have shown that the missed diagnosis rate of conventional white-light gastroscopy for gastric tumors can reach approximately 20%-30% or even higher (20). By performing real-time analysis of endoscopic images, including static images and dynamic videos, AI can assist physicians in rapidly localizing suspicious lesions and improve diagnostic accuracy and efficiency.

#### 3.2.1 Real-time detection and classification of early gastric cancer

Endoscopic image-based AI systems using convolutional neural networks (CNNs) can learn and identify subtle mucosal abnormalities associated with early gastric cancer from endoscopic images, such as slight changes in local morphology, color, and texture, by training on large volumes of annotated data, thereby helping improve the recognition of early lesions (21). In recent years, research has gradually shifted from static image analysis to real-time processing of dynamic video streams, which more closely reflects clinical examination workflows. Multicenter studies have confirmed that AI can provide real-time assisted diagnosis in magnifying image-enhanced endoscopy (M-IEE) videos (22).

In terms of lesion classification, AI models can not only effectively distinguish benign from malignant lesions, but have also shown excellent performance in image classification tasks involving gastric cancer and benign ulcers (23). Relevant reviews have further indicated that AI-based deep learning can be used to identify subtle morphological features in images of early gastric cancer and has the potential to assess lesion invasion depth and histology-related characteristics (24). Multicenter studies have shown that deep learning models perform well in fine-grained classification tasks for early gastric cancer and can assist in reducing missed diagnoses during real-time endoscopy, while improving the recognition of lesions in difficult-to-detect areas, such as the gastric fundus and the posterior wall of the gastric angle (25).

#### 3.2.2 AI analysis of magnifying endoscopy with narrow-band imaging

Magnifying endoscopy with narrow-band imaging (ME-NBI) can clearly visualize the microvascular architecture and microsurface structure of the gastric mucosa, making it an important tool for the precise diagnosis of early gastric cancer. However, it requires a high level of interpretive expertise from endoscopists (26, 27). By learning microvascular patterns, such as irregular proliferation and vascular interruption, as well as microsurface features, such as disappearance of glandular structures, from ME-NBI images, AI can assist in the precise diagnosis of early gastric cancer and the assessment of invasion depth (22, 28).

AI models based on segmentation networks such as U-Net have been applied to structural segmentation and feature extraction in endoscopic images (29). These models can automatically identify important information, including microvascular architecture and subtle surface structures, such as changes at the mucosal and lesion boundaries, thereby providing a basis for subsequent lesion classification and risk assessment. By learning these microscopic feature patterns from ME-NBI images, AI can significantly improve the diagnostic performance for early gastric cancer and has the potential to assist in clinically relevant classification tasks, such as assessing invasion depth. It is expected to reduce the learning curve for ME-NBI image interpretation and help primary care physicians perform precise early diagnosis.

### 3.3 AI-assisted applications in other imaging modalities

In addition to non-contrast CT and endoscopy, AI has also shown promising application potential in other imaging modalities. In ultrasonography, Sui et al. proposed a workflow based on oral contrast-enhanced ultrasonography (OCUS), consisting of “gastric wall detection—quantitative analysis of layered structures—screening discrimination”. In this workflow, U-Net was used to automatically localize the gastric wall region, and layered parameters, such as the relative thickness of the five-layer structure, were extracted for screening model construction. The model achieved an AUC of 0.92 in gastric disease screening, suggesting that quantitative analysis of layered structures may provide auxiliary information for noninvasive screening. However, this study was still mainly based on retrospective validation, and its performance in early lesions and cross-center generalizability requires further evaluation in larger samples (30). In PET-CT image analysis, AI can assist in determining lymph node metastasis and distant metastasis by extracting tumor metabolic and morphological features, thereby improving staging accuracy and providing a reference for treatment planning (31).

## 4 Recent advances in AI-assisted pathological diagnosis of gastric cancer

Pathological diagnosis is the “gold standard” for the definitive diagnosis of gastric cancer, with its core task being the analysis of cellular morphology, tissue architecture, and invasion status in histological sections. Conventional pathological diagnosis relies on physicians’ subjective visual assessment and is associated with limitations such as a long diagnostic turnaround time and substantial interobserver variability. The development of digital pathology has enabled the digital storage and transmission of histological slides, providing a data foundation for AI-assisted diagnosis. In recent years, the application of AI in digital pathological diagnosis of gastric cancer has covered tumor detection, histological typing and grading, assessment of invasion depth, and molecular biomarker prediction.

### 4.1 AI-assisted tumor detection and typing in digital pathology slides

Deep learning-based AI models, particularly multiple-instance learning (MIL) combined with convolutional neural networks (CNNs), can automatically analyze digitized pathological slide images, namely whole-slide images (WSIs), and extract morphological and metabolic features of tumors to assist in the diagnosis of gastric cancer and the prediction of prognosis. Multicenter retrospective studies have shown that such AI models can achieve a diagnostic accuracy of 92% for gastric cancer, approaching the performance of expert pathologists, and can provide effective prediction of overall survival. The model also performed well in external validation sets and has the potential to help clinicians accurately assess disease stage and invasion depth, thereby providing a reference for treatment planning. It is particularly suitable for large-scale screening and rapid diagnosis of early gastric cancer (32, 33).

### 4.2 AI-based assessment of invasion depth and lymph node metastasis

The depth of invasion and lymph node metastasis status of gastric cancer are key indicators for tumor staging and directly influence the selection of treatment strategies. Conventional pathological assessment is highly subjective and is prone to missing minute foci of invasion and lymph node micrometastases. By analyzing multidimensional features in whole-slide images (WSIs), AI can assist in the precise assessment of invasion depth and the efficient detection of lymph node micrometastases.

In the assessment of invasion depth, AI models can accurately predict T stage by learning features such as tumor cell invasion patterns and stromal reactions. For example, Zhu et al. used ResNet (residual network) to predict invasion depth from conventional endoscopic images, achieving an AUC of 0.94, an accuracy of 89.16%, and a specificity of 95.56%, demonstrating the excellent performance of AI in determining invasion depth (33). In the detection of lymph node metastasis, AI models have been applied to the analysis of digital pathology slides and multimodal clinical data, improving the ability to identify extremely small lesions that may be overlooked by conventional assessment.

### 4.3 AI-based prediction of molecular biomarkers using pathological images

Molecular biomarker testing, including HER2 and PD-L1, is an important basis for precision treatment of gastric cancer. Conventional testing methods, such as immunohistochemistry and fluorescence *in situ* hybridization (FISH), are associated with complex procedures, high costs, and long turnaround times. In recent years, by mining the associations between morphological features in whole-slide images (WSIs) and molecular phenotypes, AI has enabled noninvasive prediction of molecular biomarkers, providing rapid reference information for precision treatment (34).

For example, by analyzing features such as cellular morphology and the nuclear-to-cytoplasmic ratio in gastric cancer WSIs, deep learning-based AI models have achieved an accuracy of 86.7% in predicting HER2 expression status, with good concordance with immunohistochemical results (Kappa value = 0.72) (34). Existing studies have also shown that deep learning systems based on H&E-stained whole-slide images achieve relatively high performance in predicting PD-L1 expression status (AUC ≈ 0.86) (35). This technology is expected to serve as an auxiliary method for molecular biomarker testing, particularly in resource-limited primary medical institutions, where it may rapidly screen patients suitable for targeted therapy or immunotherapy. In addition, deep learning models have also shown application potential in the recognition of rare pathological subtypes of gastric cancer and in treatment decision support. Zhu et al. developed G-NECNet based on H&E-stained whole-slide images (WSIs) of gastric biopsy specimens to distinguish gastric neuroendocrine carcinoma (G-NEC) from gastric adenocarcinoma. The model achieved an AUC of 0.993 in the internal test set, 0.985 in the external single-center cohort, and 1.000 in the multicenter consultation cohort, suggesting good generalizability across different datasets. It may also reduce reliance on auxiliary tests such as immunohistochemistry to some extent (36). For signet-ring cell carcinoma (SRCC), Li et al. developed an AI model based on preoperative contrast-enhanced CT that integrated clinical features, radiomic features, and deep learning features. In the test cohort (n = 257), the model achieved an AUC of 0.786 for SRCC diagnosis. It could also perform prognostic stratification, with the high-risk group showing significantly shorter median overall survival than the low-risk group (38.8 vs. 64.2 months, p = 0.009). In patients with pathologically confirmed advanced SRCC, the model further indicated that low-risk patients derived greater benefit from adjuvant chemotherapy (37), thereby further expanding the application boundaries of pathological AI.

### 4.4 Application of unlabeled and weakly labeled data in AI-based pathological diagnosis

As AI-assisted pathological diagnosis of gastric cancer continues to evolve toward greater precision and multimodal integration, the acquisition of high-quality, fully annotated pathological data remains highly dependent on experienced pathologists (38). Owing to the complexity, time consumption, and high cost of the annotation process, the shortage of high-quality annotated data has become an important factor limiting model generalizability and clinical application. To address this issue, model training using unlabeled and weakly labeled data is becoming an important direction in digital pathology research. Semi-supervised learning combines a small number of labeled gastric cancer pathological slides with a large number of unlabeled digital slides. By using methods such as pseudo-labeling and consistency constraints, it makes fuller use of the histological information contained in unlabeled data, thereby improving the model’s ability to recognize gastric cancer and related lesions (39). Self-supervised learning, by contrast, does not rely on manual annotation. Instead, it first learns general feature representations from large-scale unlabeled pathological images and then completes task-specific training with a small amount of labeled data, thereby reducing dependence on manual annotation to some extent (40). Weakly supervised learning, in comparison, more commonly uses case-level or slide-level labels. Through multiple-instance learning and attention mechanisms, it enables classification of whole-slide images and localization of suspicious lesion regions, and has shown promising application prospects in recognition tasks involving poorly differentiated adenocarcinoma, signet-ring cell carcinoma, and other gastric cancer subtypes (41). Overall, these methods are of considerable significance for reducing the annotation burden in gastric cancer pathology, improving model practicality, and promoting clinical translation.

## 5 Multimodal data fusion-based artificial intelligence diagnostic technologies

Single-modality data can reflect only one aspect of gastric cancer and are therefore insufficient for comprehensively characterizing tumor heterogeneity. Multimodal data fusion integrates radiomics, pathological morphology, clinical information, serological indicators, and multi-omics data to construct a more comprehensive disease representation system, thereby significantly improving diagnostic accuracy and stability (42). In recent years, multimodal AI-based fusion diagnosis has become a research hotspot and has shown substantial application potential.

### 5.1 Technical architecture of multimodal data fusion

**Figure 1:**
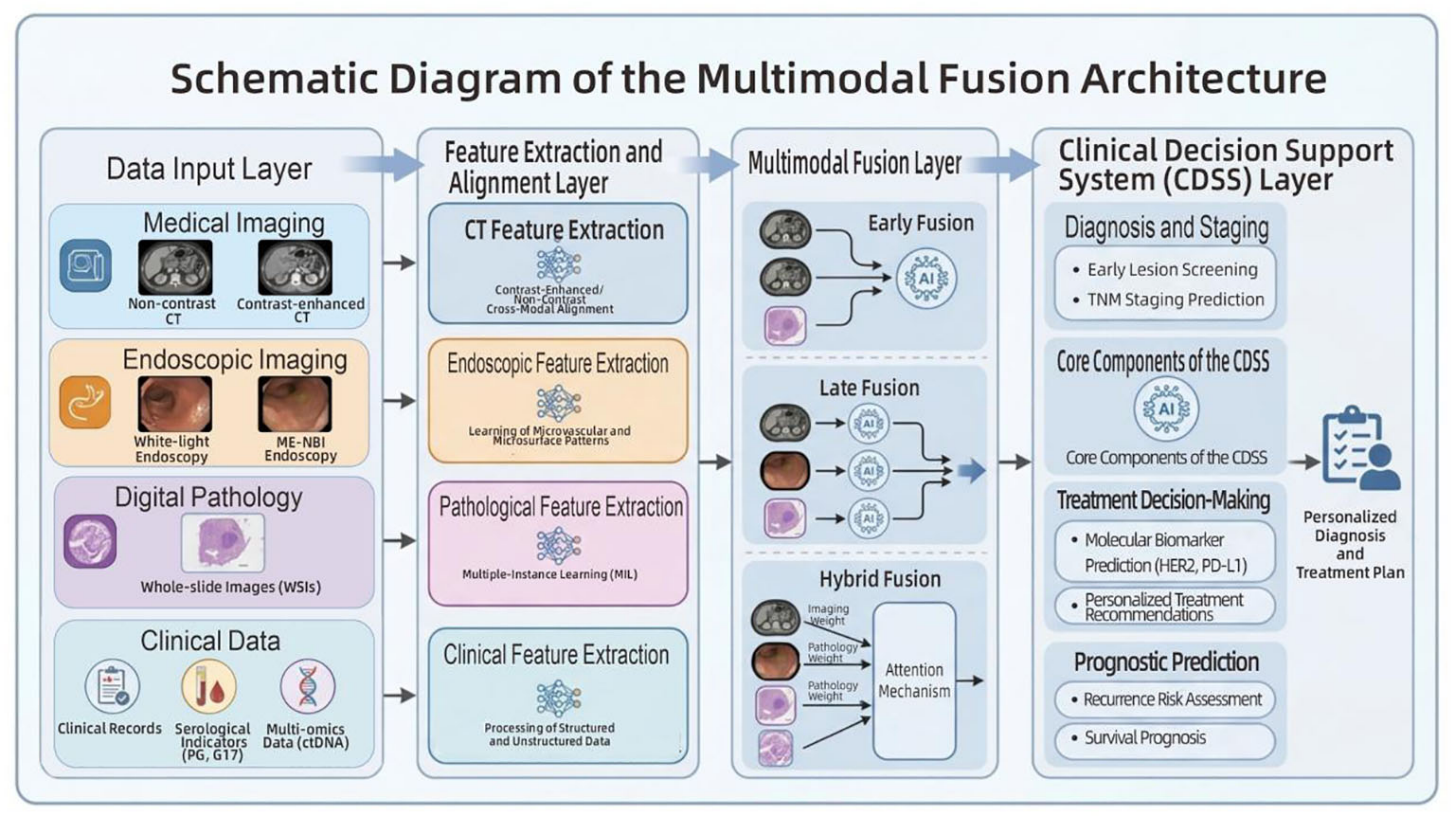
Architecture of multimodal fusion.

The core challenge of multimodal fusion lies in data heterogeneity, as imaging data are represented as pixel matrices, whereas clinical data are typically structured variables. Appropriate architectures are therefore required to achieve feature alignment and fusion. Current mainstream fusion architectures can be classified into three categories: ① early fusion, in which multimodal data are integrated during the feature extraction stage and fed into a single model for training; this approach is suitable for scenarios with similar data dimensions, such as different imaging modalities; ② late fusion, in which features are extracted and predictions are made separately for each modality, followed by fusion of the final outputs; this approach is suitable for highly heterogeneous scenarios, such as imaging data combined with clinical data; and ③ hybrid fusion, which combines the advantages of the first two approaches and dynamically adjusts the weights of different modalities through attention mechanisms to improve fusion performance (43). See **Figure 1**.

### 5.2 Implementation pathway of a CDSS for multimodal data fusion-based AI in clinical diagnosis

The key to multimodal data fusion-based AI diagnostic technology lies in integrating multisource heterogeneous information, including medical images, pathological images, clinical records, and molecular biomarkers, to overcome the limitations of single data sources and improve the accuracy of disease diagnosis and treatment decision-making (44). Among these technologies, the AI-assisted clinical decision support system (CDSS) is an important vehicle for translating multimodal AI into clinical practice (45). In general, such systems comprise four layers: data input and preprocessing, multimodal fusion, decision reasoning and knowledge integration, and output interaction. These layers are responsible for data access and standardization, feature integration, clinical adaptation, and the output of diagnostic and therapeutic recommendations, respectively (16). The generation of diagnostic and therapeutic recommendations by AI based on multimodal data is essentially a continuous process involving data fusion, feature extraction, knowledge matching, and personalized reasoning (46). This process first aligns and integrates data from different modalities. On this basis, it performs disease recognition, stratified diagnosis, and risk assessment, and subsequently combines clinical guidelines, knowledge bases, and prior cases to generate personalized recommendations. System performance is then continuously optimized through clinical feedback. Overall, multimodal CDSS promotes the deep integration of AI with clinical diagnosis and treatment and shows promising application prospects. However, further improvements are still needed in data heterogeneity, model interpretability, and generalizability.

### 5.3 Proposed multimodal AI-CDSS framework for gastric cancer diagnosis

To make the methodological pathway clearer, future AI-assisted gastric cancer studies may adopt a structured multimodal AI-CDSS framework consisting of six sequential modules. First, the data acquisition module should collect non-contrast CT, contrast-enhanced CT, white-light endoscopy, NBI or ME-NBI images and videos, H&E-stained whole-slide images, IHC slides, serological indicators, molecular biomarkers, and clinical reports. Second, the preprocessing module should standardize DICOM metadata and Hounsfield-unit normalization for CT, remove blurred or low-quality endoscopic frames, perform key-frame extraction from videos, normalize staining and tile whole-slide images, and extract structured variables from radiology, endoscopy, and pathology reports.

Third, modality-specific feature extractors should be selected according to data type: 3D CNNs, 3D ResNet, or Swin Transformer models for volumetric CT; CNN, YOLO-type detectors, or Video Transformer models for endoscopic image and video analysis; attention-based multiple-instance learning or Transformer-MIL for WSI-level pathology; gradient boosting, logistic regression, or multilayer perceptron models for structured clinical and serological data; and clinical language models for unstructured reports.

Fourth, a hybrid fusion layer with gated attention is recommended for real-world clinical data because it allows modality-specific feature extraction while dynamically weighting complementary information and tolerating missing modalities better than pure early fusion.

Fifth, the output layer should generate clinically interpretable results rather than a single binary label, including gastric cancer risk score, early gastric cancer probability, benign/malignant classification, lesion localization, T/N staging prediction, HER2 or PD-L1 prescreening, recurrence risk, and recommended next diagnostic step such as endoscopy, biopsy, ESD, surgery, follow-up, or molecular confirmation. Sixth, the validation and interpretation module should report AUC, accuracy, sensitivity, specificity, PPV/precision, NPV, F1 score, calibration, and decision-curve analysis, and should provide Grad-CAM, attention heatmaps, segmentation masks, or SHAP-based feature contributions to support clinical trust.

### 5.4 Clinical application scenarios of multimodal fusion

Multimodal fusion AI models have shown outstanding performance in early screening, precise staging, and prognostic prediction of gastric cancer. In early screening, AI models integrating non-contrast CT with serological indicators, such as PG and G17, can improve the positive predictive value of gastric cancer screening. In precise staging, multimodal fusion models have demonstrated the potential to outperform single-modality imaging assessment. For example, by integrating CT radiomics and pathological image information, these models can effectively distinguish between different TNM stage groups and between stages I-II and stage III disease (47), thereby improving preoperative assessment and treatment decision-making. In prognostic prediction, models that integrate pretreatment imaging, pathological features, molecular biomarkers, and treatment response data can accurately identify populations at high risk of recurrence and optimize postoperative adjuvant treatment strategies (48). In addition, endoscopy-based AI technologies can assist in the detection of early gastric cancer by visualizing abnormal mucosal acidity, further enriching the technical pathways for multimodal diagnosis (49).

**Figure 2:**
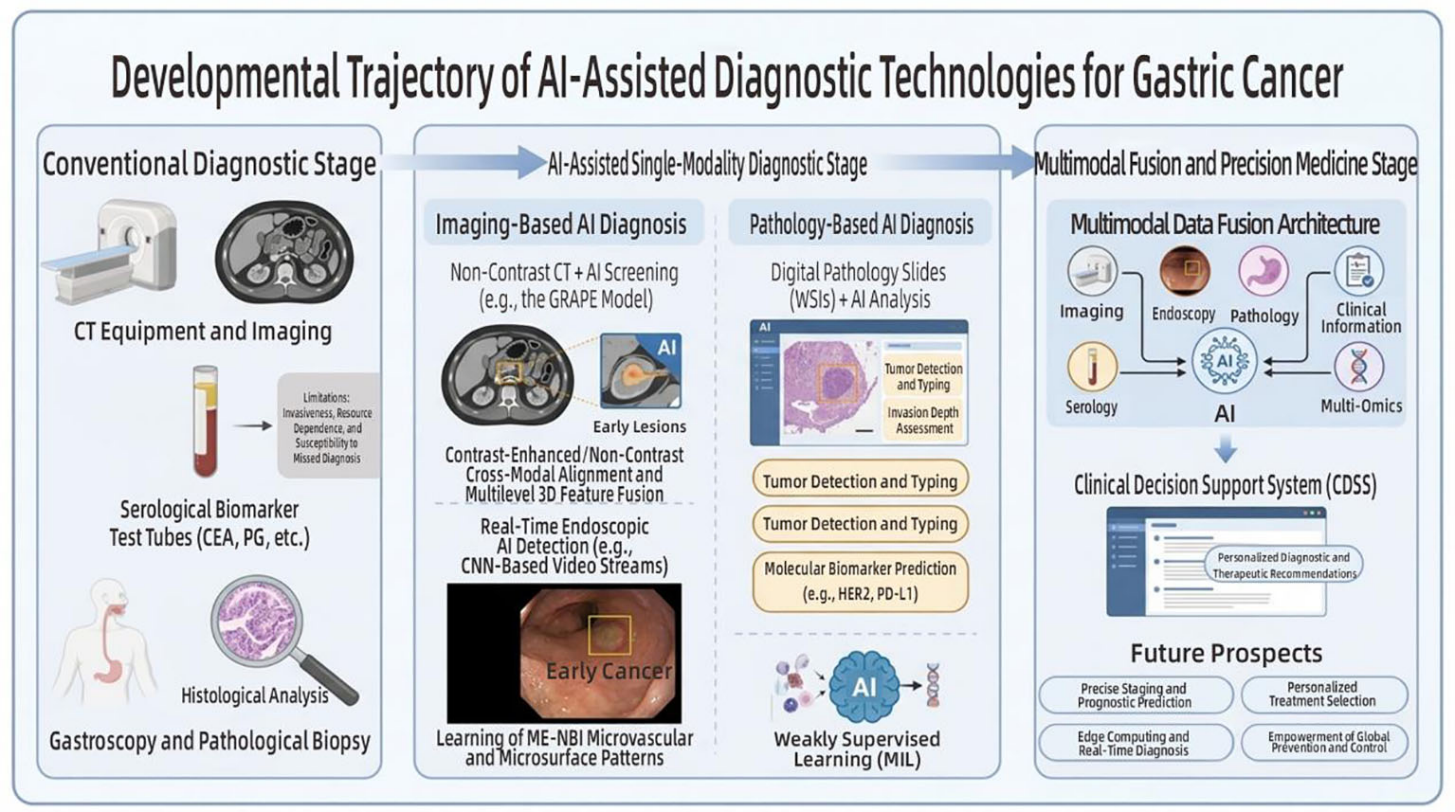
Developmental roadmap of gastric cancer diagnostic technologies.

With gastric cancer diagnostic technologies having progressed from the stage of conventional diagnosis to that of AI-assisted diagnosis, AI-assisted diagnostic technologies for gastric cancer have further evolved from single-modality analysis to deep multimodal fusion. Early studies mainly focused on feature extraction and classification using a single data type, such as imaging or pathology, with the aim of improving the efficiency and accuracy of specific diagnostic steps. With the continuous iteration of deep learning algorithms and the ongoing accumulation of high-quality medical data, the research focus has gradually shifted toward cross-modal information integration. By integrating imaging, endoscopic, pathological, and clinical information, researchers have constructed a more comprehensive disease representation system, thereby achieving an upgrade from “point-based assistance” to “whole-process empowerment”. This evolution has not only significantly improved diagnostic precision and stability, but has also laid a solid foundation for the large-scale and equitable application of AI technologies in primary medical institutions. See **Figure 2**.

## 6 Comparative performance, model selection, and data requirements for future studies

Representative studies show that AI-assisted technologies have covered screening, diagnosis, staging, molecular prediction, and prognosis in gastric cancer. However, qualitative descriptions alone are insufficient for clinical translation. A clinically useful comparison should specify the diagnostic task, data modality, model architecture, dataset size, validation design, and quantitative performance metrics. **Table 1** therefore summarizes representative studies with available AUC, accuracy, sensitivity, specificity, precision/PPV, NPV, and F1 score. Metrics not reported in the original studies are marked as NR.

**Table 1:** Comparative performance of representative AI models for gastric cancer diagnosis and related decision support.

Modality/task	Representative model or study	Architecture and data input	Dataset and validation design	Reported metrics	Recommended use and main limitation
Non-contrast CT screening	GRAPE model (18)	3D deep learning; contrast-enhanced to non-contrast cross-modal supervision; dual-branch segmentation-classification architecture.	Development cohort: 3,470 GC cases and 3,250 non-GC cases from two centers; internal validation, external validation from 16 centers, and real-world opportunistic cohorts.	Internal AUC 0.970, sensitivity 85.1%, specificity 96.8%; external AUC 0.927, sensitivity 81.7%, specificity 90.5%; real-world sensitivity 88.7%-91.9%, specificity 86.1%-95.1%; precision/PPV and F1: NR.	Best for opportunistic population screening from routine CT. Prospective PPV, false-positive burden, and cost-effectiveness still need evaluation.
White-light endoscopy/early GC detection	CNN- and video-based systems (20, 21, 25)	CNN or deep neural network models for lesion localization, blind-spot control, and real-time video-frame analysis.	Mostly retrospective image datasets and selected prospective/tandem endoscopy studies; multicenter validation remains uneven across studies.	Meta-analysis of endoscopic AI for early GC: pooled sensitivity 0.86, specificity 0.90, AUC 0.94; WLI subgroup sensitivity 0.73, specificity 0.89, AUC 0.90; precision/PPV and F1 often NR.	Useful for reducing missed lesions during routine gastroscopy. Performance depends on image quality, lesion size, lesion location, and operator technique.
ME-NBI/fine-grained early GC diagnosis	ENDOANGEL-ME and ME-NBI AI systems (22, 28)	Deep learning models using ME-NBI microvascular and microsurface patterns; real-time image-enhanced endoscopy video analysis.	M-IEE images from multiple hospitals; image-level, video-level, and prospective clinical evaluation have been reported.	Reported diagnostic accuracy around 90% in representative studies; sensitivity and specificity have been reported near 93% and 94% in real-time ME-NBI summaries; PPV/NPV/F1 should be reported consistently in future studies.	Best for differentiating early GC from gastritis or benign lesions under magnifying endoscopy. Requires high-quality ME-NBI images and cross-device validation.
Oral contrast-enhanced ultrasound screening	OCUS workflow with U-Net (30)	U-Net gastric-wall localization followed by quantitative analysis of layered gastric-wall structures.	Retrospective validation; cross-center and early-lesion validation still limited.	AUC 0.92 for gastric disease screening; precision/PPV, NPV, F1: NR.	Potential noninvasive adjunct for screening. Needs larger prospective cohorts and standardized acquisition protocols.
Digital pathology/tumor detection and typing	MIL + CNN, GastroMIL-type WSI analysis (32, 33)	Multiple-instance learning combined with CNN-based feature extraction from whole-slide images.	Multicenter retrospective cohorts with external validation; slide-level labels reduce annotation burden.	Diagnostic accuracy approximately 92% in external validation; AUC, sensitivity, specificity, precision/PPV, and F1 should be reported by subtype and stage.	Useful for pathological prescreening and review. Vulnerable to staining, scanning, and center-related domain shifts.
Invasion-depth assessment	ResNet-based image model (33)	Residual network learning invasion-related visual patterns from endoscopic images.	Retrospective validation; stability in real-world endoscopy settings remains to be confirmed.	AUC 0.94; accuracy 89.16%; specificity 95.56%; sensitivity, precision/PPV, and F1: NR in the current manuscript.	Supports preoperative decision-making between ESD and surgery. Needs prospective validation and transparent failure analysis.
Molecular biomarker prediction/HER2	HER2Net or WSI-based DL (34)	Weakly supervised WSI model mining morphological-molecular associations from H&E images.	Retrospective multicenter WSI cohorts; IHC/FISH remain reference standards.	Accuracy 86.7%; Kappa 0.72 in the current manuscript; precision/PPV, NPV, and calibration should be reported for clinical triage.	Potential prescreening before IHC/FISH. Cannot replace gold-standard molecular testing.
Molecular biomarker prediction/PD-L1	H&E WSI-based DL (35)	Deep learning model predicting PD-L1 expression status from H&E whole-slide images.	Preliminary evidence; the cited study is a preprint in the current reference list.	AUC approximately 0.86; accuracy, sensitivity, specificity, precision/PPV, and F1 require peer-reviewed confirmation.	May accelerate immunotherapy candidate screening. Evidence level is currently lower than that of peer-reviewed multicenter studies.
Rare subtype and prognosis/G-NEC and SRCC	G-NECNet (36); CT-based SRCC model (37)	WSI-based subtype classification for G-NEC; CT radiomics plus deep learning and clinical features for SRCC diagnosis/prognosis.	Internal, external, and multicenter consultation cohorts for G-NECNet; SRCC model tested in a cohort of 257 patients.	G-NECNet AUC: 0.993 internal, 0.985 external, 1.000 multicenter consultation; SRCC model AUC 0.786; OS stratification 38.8 vs 64.2 months.	Useful for rare subtype recognition and risk stratification. Needs broader external validation and calibration across histological subtypes.

AUC, area under the receiver operating characteristic curve; PPV, positive predictive value; NPV, negative predictive value; F1, harmonic mean of precision and recall; NR, not reported in the cited source or not available in the current manuscript.

### 6.1 Model selection and optimal alternatives for different diagnostic scenarios

No single AI architecture is universally optimal for all gastric cancer diagnostic tasks. The most appropriate model depends on the data modality, annotation granularity, clinical purpose, and deployment scenario. For opportunistic population screening using non-contrast CT, a three-dimensional segmentation-classification framework with cross-modal supervision is preferable because it can learn subtle volumetric gastric-wall morphology. For real-time endoscopic diagnosis, lightweight CNN-, YOLO-, or Transformer-based video models with temporal aggregation and quality-control modules are preferable because they can provide frame-level lesion alerts during examination. For whole-slide pathology, weakly supervised or attention-based multiple-instance learning is more suitable because slide-level labels are easier to obtain than pixel-level annotations. For comprehensive risk stratification, hybrid multimodal fusion models integrating CT, endoscopy, WSI, serological indicators, and clinical reports may offer better robustness than single-modality models, particularly when missing data are handled by modality dropout, gated attention, or late-fusion fallback strategies.

### 6.2 Data size, diversity, and impact on multimodal model performance

Data size directly affects model generalizability. Small single-center datasets increase overfitting and produce unstable sensitivity, especially for rare but clinically important targets such as T1/T2 early gastric cancer, signet-ring cell carcinoma, gastric neuroendocrine carcinoma, lymph-node micrometastasis, and HER2/PD-L1-positive subgroups. Therefore, future studies should report not only the number of patients but also the number of images, videos, WSIs, annotated lesions, positive cases, negative controls, centers, scanners, endoscopes, staining batches, and clinical subgroups.

Data diversity is equally important. CT datasets should include different vendors, slice thicknesses, reconstruction kernels, contrast phases, gastric filling states, lesion locations, and T stages. Endoscopy datasets should include WLI, NBI, ME-NBI, different endoscope systems, different operators, multiple anatomical sites, blurred frames, mucus, bleeding, and difficult-to-observe areas. Pathology datasets should cover biopsy and surgical specimens, H&E and IHC slides, staining variation, scanner variation, magnification differences, Lauren classification, differentiation grade, and rare subtypes. Clinical data should include demographic factors, symptoms, Helicobacter pylori status, family history, serum PG I/II, G17, CEA, CA724, molecular biomarkers, treatment, recurrence, and survival.

Class imbalance and missing modalities can substantially distort model performance. If early gastric cancer cases are underrepresented, a model may achieve high overall accuracy but poor sensitivity for early lesions. Therefore, accuracy alone is insufficient; future reports should include sensitivity, specificity, precision/PPV, NPV, F1 score, AUPRC, calibration, and subgroup performance. In real-world multimodal datasets, not every patient has complete CT, endoscopy, pathology, serological, and molecular data. Early-fusion models are sensitive to missing inputs, whereas late-fusion and hybrid gated-attention models are more practical for heterogeneous clinical workflows.

### 6.3 Recommended data structure for future multimodal AI studies

For transparent reporting, future studies should also include a data dictionary, preprocessing code or protocol, training/validation/test split information, center-level distribution, missing-modality rate, annotation guidelines, and subgroup performance by tumor stage, lesion location, scanner, endoscope, staining batch, age, and sex. The recommended data structure for future multimodal AI studies in gastric cancer diagnosis is summarized in **Table 2**.

**Table 2:** Recommended data structure for future multimodal AI studies in gastric cancer diagnosis.

Data type	Required raw data	Key parameters	Labels and outcomes	Related reports
CT imaging	Non-contrast CT, contrast-enhanced CT, DICOM files	Scanner vendor, slice thickness, kVp, mAs, reconstruction kernel, contrast phase, gastric distension status, lesion location	GC/non-GC, early/advanced GC, T/N/M stage, tumor location, pathology-confirmed diagnosis	Radiology report, surgical record, pathological report
Endoscopy	WLI images, NBI images, ME-NBI images, full procedure videos	Frame rate, resolution, endoscope model, anatomical site, image-quality score, blind-spot record, bowel/stomach preparation quality	Lesion bounding box, segmentation mask, benign/malignant label, invasion depth, histological diagnosis, missed-lesion status	Endoscopy report, biopsy report, procedure quality-control report
Digital pathology	H&E WSI, IHC slides, biopsy and surgical specimens	Scanner type, magnification, staining protocol, tile size, tissue area, tumor-cell percentage, batch information	Tumor region, histological subtype, differentiation grade, Lauren type, HER2, PD-L1, MSI, EBV, lymphovascular invasion	Pathology report, IHC/FISH report, molecular report
Clinical and serological data	Demographics, symptoms, risk factors, laboratory tests	Age, sex, H. pylori status, family history, smoking, alcohol, PG I/II, G17, CEA, CA724, hemoglobin, albumin	Risk category, final diagnosis, treatment choice, eligibility for endoscopy, biopsy, ESD, surgery, or systemic therapy	Admission record, discharge summary, laboratory report
Treatment and follow-up	Therapeutic records and longitudinal outcomes	ESD, surgery, chemotherapy, immunotherapy, targeted therapy, recurrence date, survival time, adverse events	Overall survival, disease-free survival, recurrence, response to therapy, treatment-related toxicity	Operative note, oncology record, follow-up record

### 6.4 Validation, reporting, and interpretability requirements

A robust AI study should include internal validation, independent external validation, and prospective clinical evaluation whenever possible. For diagnostic tasks, the minimum reporting set should include AUC, accuracy, sensitivity, specificity, precision/PPV, NPV, F1 score, calibration curve, confidence intervals, and decision-curve analysis. For segmentation tasks, Dice coefficient and intersection-over-union should be reported. For clinical deployment, model outputs should be interpretable through heatmaps, segmentation masks, attention maps, or feature-contribution analyses, and the threshold used for clinical referral should be prespecified according to the intended scenario, such as high-sensitivity screening or high-specificity diagnostic confirmation.

## 7 Challenges and countermeasures in AI-assisted diagnosis of gastric cancer

Despite the significant progress achieved by AI-assisted technologies, their clinical translation and large-scale application still face multiple challenges, including data quality, model interpretability, ethical standards, and adaptation to healthcare systems. Researchers and industry stakeholders have undertaken a series of explorations and proposed corresponding countermeasures.

### 7.1 Data quality and standardization

The performance of AI models is highly dependent on the quality and quantity of training data. The main data-related challenges currently include: ① data heterogeneity: substantial differences in imaging equipment models and parameter settings across institutions may lead to inconsistent lesion appearances and reduce model generalizability (50); ② variable annotation quality: annotations by specialist physicians are inherently subjective, which may affect training performance (51); and ③ data scarcity: early gastric cancer cases are relatively rare, making it difficult to construct large-scale, balanced datasets.

More specifically, insufficient sample size primarily increases overfitting and reduces the stability of sensitivity in rare early-stage lesions. Limited data diversity causes domain shift when models are applied to images from unseen CT scanners, endoscopy systems, staining protocols, or patient populations. In multimodal models, incomplete modalities may reduce performance if the fusion architecture cannot handle missing inputs. Therefore, future studies should report the number of patients, number of images or WSIs, number of centers, tumor-stage distribution, device types, annotation granularity, and missing-modality rate.

The main countermeasures include the following: ① promoting data standardization. Data standardization is an important approach to improving the cross-center generalizability of medical AI models. Studies have shown that, compared with nonstandardized data, the use of consistent acquisition protocols, preprocessing workflows, and feature alignment methods can significantly reduce differences in data distribution across institutions and devices, thereby alleviating domain shift and improving the stability and accuracy of AI models on data from external institutions (52); ② constructing multicenter collaborative datasets. For example, the DAMO GRAPE dataset covers 20 medical centers and significantly improves model generalizability (18); and ③ introducing semi-supervised and weakly supervised learning. Training with a small amount of labeled data and a large amount of unlabeled data can help alleviate the scarcity of early gastric cancer data (53).

### 7.2 Ethical standards and regulation

The core ethical and regulatory challenges include the following: ① unclear allocation of responsibility: if AI fails to identify early gastric cancer and thereby delays treatment, it remains unclear whether responsibility should be borne by the AI developer, the medical institution, or the physician; ② risks to data privacy and security: AI models for gastric cancer diagnosis rely on large volumes of patient data, including CT and endoscopic data, and if such data are stored or processed through third-party cloud services, there may be a risk of data leakage; ③ algorithmic bias: population bias in training data may reduce diagnostic performance in specific populations, leading to healthcare inequity; and ④ regulatory lag: policy updates may struggle to keep pace with the rapid development of technology.

Countermeasures include the following: ① improving laws and regulations by defining AI as an “auxiliary tool”, with physicians bearing the ultimate responsibility for diagnosis; ② implementing strict data security measures, such as requiring real-time audit logs and localized data storage; ③ strengthening the monitoring of algorithmic fairness by requiring model performance validation across different populations and quarterly updates of subgroup performance curves; and ④ establishing a dynamic regulatory framework by implementing a tiered certification system for AI medical products to enable ongoing supervision.

Against this background, it is noteworthy that China’s National Medical Products Administration (NMPA) has approved several AI-assisted diagnostic products for gastric cancer, including AI-assisted diagnostic systems for gastrointestinal endoscopy and pathological AI analysis software. For example, EndoScreener developed by Wision Medical is the first gastrointestinal endoscopy AI product in China to obtain NMPA Class III medical device approval. It can perform real-time dynamic analysis of endoscopic examination images and alert physicians to suspicious lesions, thereby assisting doctors in improving the detection rate of gastrointestinal tumors, including early gastric cancer. The product has also received multiple international certifications, including clearance from the US Food and Drug Administration (FDA) and certification under the European Union Medical Device Regulation (CE-MDR), strengthening confidence in its international clinical application and regulatory acceptance.

## 8 Future prospects

With the development of AI technologies and the evolution of clinical needs, AI-assisted diagnosis of gastric cancer will move toward greater precision, efficiency, and accessibility. Future breakthroughs are expected in the following areas:

### 8.1 Deep enhancement of multimodal fusion

Further integration of radiomics, pathological morphology, multi-omics data, wearable device data, and clinical information will enable the construction of a more comprehensive disease representation system. For example, the integration of dynamic ctDNA methylation monitoring (54) with conventional imaging may realize the integration of early screening and recurrence warning.

### 8.2 Precision medicine and personalized diagnosis

AI will be deeply integrated into precision medicine systems to provide personalized diagnostic and therapeutic recommendations by analyzing individual patient characteristics, such as genetic background and pathological subtype. For example, AI-based molecular biomarker prediction models can rapidly screen patients suitable for targeted therapy or immunotherapy and dynamically adjust diagnostic and treatment strategies (55).

### 8.3 Edge computing and real-time diagnosis

With the widespread application of edge computing, lightweight AI models can be deployed on medical devices, such as endoscopes and CT scanners, to enable real-time analysis of imaging data and immediate feedback, thereby reducing transmission latency and privacy risks (56). For example, real-time AI diagnostic modules integrated into endoscopic devices can immediately alert physicians to suspicious lesions and assist in precise endoscopic operation. Evidence from other AI-assisted screening and computational pathology applications, including pulmonary nodule risk assessment, autonomous diabetic retinopathy detection, and weakly supervised whole-slide image analysis, further supports the feasibility of scalable clinical deployment (57–60).

### 8.4 AI empowerment for global gastric cancer prevention and control

AI provides new solutions for global gastric cancer prevention and control, particularly in developing countries with limited medical resources. Low-cost and broadly applicable screening models, such as “non-contrast CT + AI”, can rapidly improve the rate of early diagnosis. The DAMO GRAPE model developed in China provides a localized solution for noninvasive gastric cancer screening, and its low-cost and highly generalizable features offer a new technical reference for global gastric cancer prevention and control.

## 9 Conclusion

Artificial intelligence-assisted technologies have achieved a series of breakthrough advances in gastric cancer diagnosis, demonstrating significant clinical value in non-contrast CT-based opportunistic screening, endoscopic image and video analysis, digital pathological diagnosis, molecular biomarker prediction, and multimodal fusion. Compared with the original single-modality paradigm, future AI systems should move toward standardized multicenter datasets, transparent model comparison, hybrid fusion architectures, clinically interpretable outputs, and prospective validation. AI models represented by GRAPE illustrate the potential of noninvasive and efficient screening, while WSI-based MIL models and endoscopy-based real-time systems show the value of AI in pathological confirmation and lesion detection. However, clinical translation still requires rigorous evaluation of sensitivity, specificity, precision/PPV, NPV, F1 score, calibration, subgroup fairness, privacy protection, and regulatory compliance. With the continued development of multimodal fusion, precision medicine, and edge deployment, AI-assisted gastric cancer diagnosis is expected to become a key enabling technology for early detection, individualized decision-making, and global gastric cancer prevention and control.
